# Bis(μ-*N*,*N*-di­allyl­dithio­carbamato)bis[(*N*,*N*-di­allyl­dithio­carbamato)cadmium]

**DOI:** 10.1107/S2056989017011616

**Published:** 2017-08-21

**Authors:** Damian C. Onwudiwe, Madalina Hrubaru, Eric C. Hosten, Charmaine Arderne

**Affiliations:** a Department of Chemistry, School of Mathematical and Physical Sciences Faculty of Agriculture, Science and Technology, North-West University (Mafikeng Campus), Private Bag X2046, Mmabatho 2735, South Africa; b Material Science Innovation and Modelling (MaSIM) Research Focus Area, Faculty of Agriculture, Science and Technology, North-West University (Mafikeng Campus), Private Bag X2046, Mmabatho, South Africa; c C.D. Nenitescu Center of Organic Chemistry of the Romania Academy, Splaiul Independentei, 2023, Bucharest, Romania; d Department of Chemistry, Nelson Mandela Metropolitan University, PO Box 77000, Port Elizabeth 6031, South Africa; e Department of Chemistry, University of Johannesburg, PO Box 524, Auckland Park, Johannesburg 2006, South Africa

**Keywords:** crystal structure, cadmium(II) complex, *N*,*N*-di­allyl­ldi­thio­carbamate ligands, bridging dimeric structure

## Abstract

The title compound, [Cd_2_(C_7_H_10_NS_2_)_4_], is a neutral dinuclear cadmium(II) complex bearing four bis *N*,*N*-di­allyl­di­thio­carbamate ligands coordinating to two Cd^II^ cations. In each of the monomeric subunits, there are four S atoms of two di­thio­carbamate ligands [Cd—S = 2.5558 (3), 2.8016 (3), 2.6050 (3) and 2.5709 (3) Å] that coordinate to one Cd^II^ atom in a bidentate mode. The dimers are located over an inversion centre bridged by two additional bridging Cd—S bonds [2.6021 (3) Å], leading to a substantial distortion of the geometry of the monomeric subunit from the expected square-planar geometry. The five-coordinate environment around each of the Cd^II^ ions in the dimer is best described as substanti­ally tetra­gonally distorted square pyramidal. The di­thio­carbamate groups are themselves planar and are also coplanar with the Cd^II^ ions. The negative charge on these groups is delocalized by resonance across the S atoms bound to the Cd^II^ cation. This delocalization of the π electrons in the di­thio­carbamate groups also extends to the C—N bonds as they reveal significant double bond character [C—N = 1.3213 (16) and 1.3333 (15) Å].

## Abstract 2

The characteristic feature of this cadmium(II) complex is the formation of a dimeric bridged structure where the two Cd^II^ cations are bridged by S atoms from the *N*,*N*-di­allyl­ldi­thio­carbamate ligands.

## Chemical context   

Inter­est in the study of metal di­thio­carbamates was aroused because of their inter­esting structural features and diverse applications (Thammakan & Somsook, 2006 ▸). Di­thio­carbamate complexes have largely been prepared from the group 12 elements, mostly because they have found wide practical application as additives to pavement asphalt, as anti­oxidants, and as potent pesticides *etc* (Subha *et al.*, 2010 ▸). The structural chemistry of cadmium di­thio­carbamates of the general formula Cd(S_2_CN*RR*′) where *R*, *R*′ = alkyl or aryl is dominated by its existence in binuclear form. This common feature has been ascribed to the effect of aggregated species, which they adopt in the solid state, resulting from equal numbers of μ_2_-tridentate and bidentate (chelating) ligands (Tiekink, 2003 ▸; Tan, Halim *et al.*, 2016 ▸). Only a few exceptions have been reported where the complex exists in a trinuclear form (Kumar *et al.*, 2014 ▸), or as a one-dimensional polymeric motif (Tan *et al.*, 2013 ▸, 2016 ▸; Ferreira *et al.*, 2016 ▸). Bis­(*N*,*N*-di­allyl­di­thio­carbamato)cadmium compounds have the advantage of having stability similar to that of the zinc complexes, but more favourable stability when compared to the mercury complexes. Cadmium di­thio­carbamate complexes have been widely used as single-source precursors for CdS nanoparticles and thin films, which have application as non-linear optical materials (Thammakan & Somsook, 2006 ▸). Another important practical application of cadmium di­thio­carbamates is their ability to efficiently collect gold from acidic solutions (Rodina *et al.*, 2014 ▸). Here we describe the crystal structure of a Cd^II^ complex bearing a di­allyl­dithio­carabamate ligand in a chelating and bridging dimeric structure.
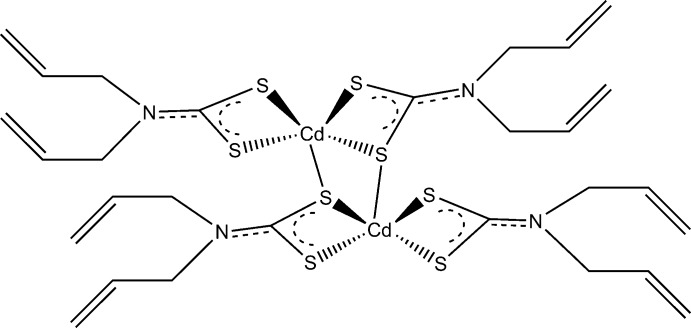


## Structural commentary   

**Figure 1:**
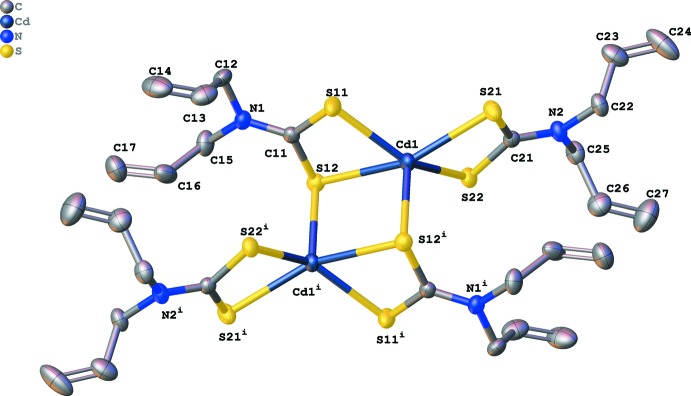
The mol­ecular structure of the title compound, showing 50% probability displacement ellipsoids and the atomic numbering scheme [symmetry code: (i) −*x* + 2, −*y*, −*z* + 1]. H atoms have been omitted for clarity.

The coordination environment of the Cd^II^ cation is observed to have a distorted tetra­gonal–pyramidal geometry (Fig. 1 ▸). The Cd^II^ cation is coordinated by four S atoms with distances ranging from 2.5558 (3) to 2.8016 (3) Å and to a fifth S atom at a distance of 2.6021 (3) Å; these distances are similar to other complexes found to have been published previously (see Section 4: *Database survey*). A full geometry check carried out with the *Mogul* Geometry Check tool (Bruno *et al.*, 2004 ▸) within the CSD suite of programs, showed no unusual geometrical parameters. The fifth S atom, S12^i^, is from a third ligand that is in the coordination sphere of a centrosymmetrically related Cd^II^ ion [symmetry code: (i) –*x* + 2, –*y*, –*z* + 1]. This means that each bridging S atom simultaneously occupies an equatorial coordination site on one Cd^II^ ion and an apical site on the other Cd^II^ ion to form an edge-shared tetra­gonal–pyramidal geometry. The Cd^II^ ion deviates from the S11—S12—S22—S21 mean plane by 0.704016 (17) Å towards S12^i^. The bridging network Cd1—S12—Cd1^i^—S12^i^ is completely planar since it lies over the inversion centre with a Cd1⋯Cd1^i^ separation distance of 3.60987 (8) Å and S12—Cd1—S12^i^ and Cd1—S12—Cd1^i^ angles of 96.257 (9) and 83.743 (9)°, respectively. There is substantial distortion of the geometry of the monomeric subunit from the expected square-planar geometry. Deviations from the standard 90° angles are evident in the angles of S11—Cd1—S21 [108.203 (11)°]; S22—Cd1—S21 [70.264 (10)°]; S22—Cd1—S12 [96.950 (10)°] and S11—Cd1—S12 [67.486 (10)°]. Deviations in the standard 180° angles are evident in the angles of S11—Cd1—S22 [143.705 (13)°] and S21—Cd1—S12 [152.651 (11)°]. The Cd1—S12—Cd1^i^—S12^i^ and S11—S12—S22—S21 mean planes form a dihedral (twist) angle of 84.6228 (18)°. The di­thio­carbamate groups are planar and each group of the monomeric subunit is coplanar with the Cd^II^ ion (r.m.s. deviation is 0.010 Å). The mean plane consisting of atoms Cd1, S11, N1, C11, S12 and the mean plane consisting of atoms Cd1, S22, N2, C21, S21 have a plane-normal-to-plane-normal angle of 37.0291 (10)°; a centroid-to-centroid distance of 4.45354 (8) Å; a plane-to-plane shift of 4.22298 (8) Å and a plane-to-plane torsion (twist) angle of 8.0304 (12)°.

The S12—C11 bond length [1.7532 (13) Å] is longer than the adjacent S11—C11 bond length [1.7162 (13) Å] suggesting that this bond has more double bond character in the di­thio­carbamate portion that coordinates to the Cd^II^ cation. On the opposite side of the Cd^II^ ion, both S—C bonds have approximately the same length, where S21—C21 and S22—C21 bond lengths are 1.7224 (12) and 1.7263 (12) Å, respectively, suggesting that the double bond of the di­thio­carbamate is spread over the S—C—S bond *via* resonance. A possible explanation for this may be because of the fact that atom S12 serves as the bridging S atom in the complex. Also, the N1—C11 and N2—C21 distances [1.3213 (16) and 1.3333 (15) Å, respectively] are shorter compared to the other N—C distances indicating considerable double-bond character. The vinyl substituents are also planar and are at an angle of 91.6049 (14)° from the di­thio­carbamate plane and at an angle of 150.9196 (6)° from the vinyl group directly opposite from it. This scenario is comparable with the other structures surveyed in the literature (see Section 4: *Database survey*). All highlighted and discussed geometrical parameters describing the coordination environment are given in Table 1 ▸. Weak intramolecular C—H⋯S inter­actions are observed (Table 2 ▸)

## Supra­molecular features   

**Figure 2:**
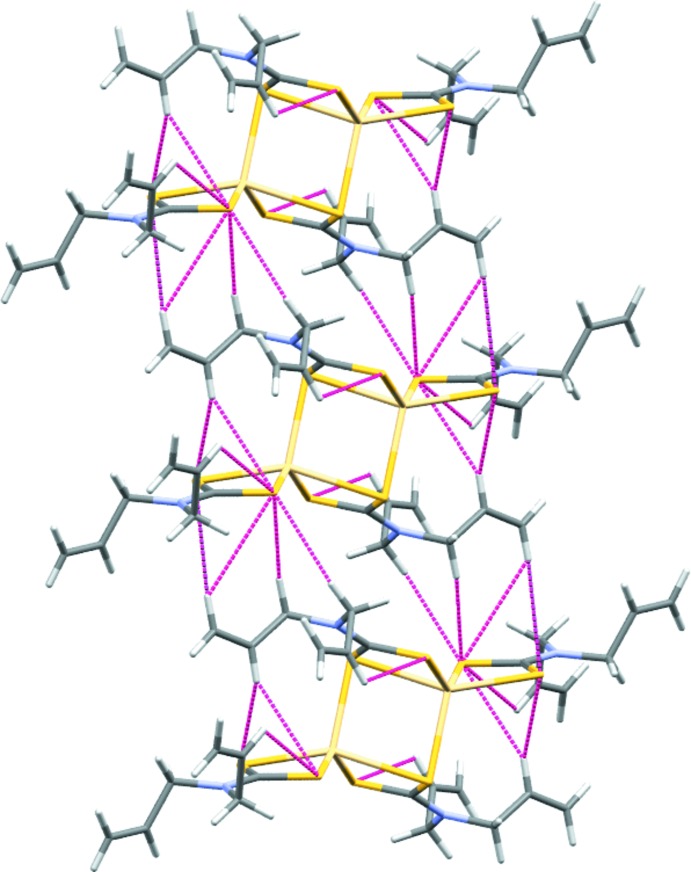
The crystal structure of the title compound constructed from chains formed by C—H⋯S inter­actions (red dashed lines). **[Authors: Please add unit cell outline and coordinate axes]**

The space group of the crystal is *P* 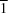, and the asymmetric unit consists of one-half of the complex mol­ecule, so that the unit cell contains one complete complex mol­ecule. Each half of the asymmetric unit is related by an inversion centre. In the crystal, weak C—H⋯π inter­actions are observed, forming chains along [001] (see Fig. 2 ▸ and Table 3 ▸).

## Database survey   

A search of the Cambridge Structural Database (version 1.19, May 2017 updates) (Groom *et al.*, 2016 ▸) revealed that there are a number of similar types of compounds where in place of the *N*,*N*-diallyl side chain, the side-chains substituents are di-*n*-propyl [CSD refodes BEHNOR (Jian *et al.*, 1999*a* ▸), BEHNOR01 (Ivanov *et al.*, 2005 ▸)], di-isobutyl [LESVEK (Cox & Tiekink, 1999 ▸), LESVEK01 (Glinskaya *et al.*, 1999 ▸)] and di-isopropyl [SUVTUY (Jian *et al.*, 1999*b* ▸), SUVTUY01 (Cox & Tiekink, 1999 ▸)].

## Synthesis and crystallization   

A solution of CdCl_2_·2H_2_O (0.55 g, 0.0025 mol) in ethanol (10 ml) was added to a solution of sodium *N*,*N*-diallyl di­thio­carbamate (0.98 g, 0.005 mol) in ethanol (10 ml), and the resulting suspension was stirred for 45 min at room temperature. This solution was then filtered, and rinsed several times with distilled water (Onwudiwe *et al.*, 2015 ▸) and ethanol. Yield: 1.28 g, 56%. Analysis found: C, 36.38; H, 4.40; N, 6.50; S, 28.42%. Calculated for C_14_H_20_N_2_S_4_Cd: C, 36.79; H, 4.41; N, 6.13; S, 28.06. Crystals suitable for single-crystal X-ray analysis were obtained by recrystallization from chloro­form/ethanol. Other analytical data for this material (melting point, IR and NMR data) has been published previously (Onwudiwe *et al.*, 2015 ▸).

## Refinement   

Crystal data, data collection and structure refinement details are summarized in Table 4 ▸. All H atoms were positioned geometrically and refined isotropically using the riding-model approximation with C—H = 0.99 Å and *U*_iso_(H) = 1.2 *U*_eq_(C) for methyl­ene groups and C—H = 0.95 Å and *U*_iso_(H) = 1.2 *U*_eq_(C) for all vinyl groups.

## Supplementary Material

Crystal structure: contains datablock(s) I. DOI: 10.1107/S2056989017011616/zl2710sup1.cif

Structure factors: contains datablock(s) I. DOI: 10.1107/S2056989017011616/zl2710Isup2.hkl

Supporting information file. DOI: 10.1107/S2056989017011616/zl2710Isup3.mol

CCDC reference: 899314

Additional supporting information:  crystallographic information; 3D view; checkCIF report

## Figures and Tables

**Table 1:** Selected geometric parameters (Å, °)

Cd1—S11	2.5558 (3)	S12—C11	1.7532 (13)
Cd1—S22	2.5709 (3)	S21—C21	1.7224 (12)
Cd1—S12^i^	2.6021 (3)	S22—C21	1.7263 (12)
Cd1—S21	2.6050 (3)	N1—C11	1.3213 (16)
Cd1—S12	2.8016 (3)	N2—C21	1.3333 (15)
S11—C11	1.7162 (13)		
			
S11—Cd1—S22	143.705 (13)	S22—Cd1—S12	96.950 (10)
S11—Cd1—S21	108.203 (11)	S12^i^—Cd1—S12	96.257 (9)
S22—Cd1—S21	70.264 (10)	S21—Cd1—S12	152.651 (11)
S11—Cd1—S12	67.486 (10)	Cd1^i^—S12—Cd1	83.743 (9)

Symmetry code: (i) 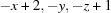.

**Table 2:** Hydrogen-bond geometry (Å, °)

*D*—H⋯*A*	*D*—H	H⋯*A*	*D*⋯*A*	*D*—H⋯*A*
C12—H12*B*⋯S11	0.99	2.59	2.9783 (14)	103
C15—H15*A*⋯S12	0.99	2.50	3.0438 (14)	115
C22—H22*B*⋯S21	0.99	2.50	3.0381 (13)	114
C25—H25*B*⋯S22	0.99	2.56	2.9845 (14)	106

**Table 3:** *X*—H⋯π inter­actions *Cg*3 is the centroid of the Cd1—S11—C11—S12—Cd1^i^—S12^i^ ring.

C—H⋯*Cg*	C—H	H⋯*Cg*	C⋯*Cg*	C—H⋯*Cg*
C15—H15*B*⋯*Cg*3	0.99	2.94	3.9209 (17)	171
C16—H16⋯*Cg*3	0.99	2.90	3.7648 (17)	152

Symmetry code: (i) −*x* + 2, −*y*, −*z* + 1.

**Table 4:** Experimental details

Crystal data	
Chemical formula	[Cd_2_(C_7_H_10_NS_2_)_4_]
*M*_r_	913.92
Crystal system, space group	Triclinic, *P* 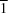
Temperature (K)	200
*a*, *b*, *c* (Å)	8.0872 (2), 9.4146 (2), 13.0721 (3)
α, β, γ (°)	75.858 (1), 78.460 (1), 77.488 (1)
*V* (Å^3^)	930.75 (4)
*Z*	1
Radiation type	Mo *K*α
μ (mm^−1^)	1.62
Crystal size (mm)	0.60 × 0.44 × 0.17

Data collection	
Diffractometer	Bruker APEXII CCD
Absorption correction	Numerical (*SADABS*; Bruker, 2011 ▸)
*T*_min_, *T*_max_	0.824, 1.000
No. of measured, independent and observed [*I* > 2σ(*I*)] reflections	16101, 4644, 4391
*R*_int_	0.015
(sin θ/λ)_max_ (Å^−1^)	0.669

Refinement	
*R*[*F* ^2^ > 2σ(*F* ^2^)], *wR*(*F* ^2^), *S*	0.015, 0.037, 1.15
No. of reflections	4644
No. of parameters	191
H-atom treatment	H-atom parameters constrained
Δρ_max_, Δρ_min_ (e Å^−3^)	0.28, −0.31

Computer programs: *BIS* and *APEX2* (Bruker, 2011 ▸), *SAINT* (Bruker, 2009 ▸), *SHELXS97* (Sheldrick, 2008 ▸), *SHELXL2017* (Sheldrick, 2015 ▸), *ORTEP-3 for Windows* (Farrugia, 2012 ▸), *PLATON* (Spek, 2009 ▸) and *Mercury* (Macrae *et al.*, 2008 ▸).

## supplementary crystallographic information

### Crystal data

**Table:**

[Cd_2_(C_7_H_10_NS_2_)_4_]	*Z* = 1
*M**_r_* = 913.92	*F*(000) = 460
Triclinic, *P*1	*D*_x_ = 1.631 Mg m^−^^3^
*a* = 8.0872 (2) Å	Mo *K*α radiation, λ = 0.71073 Å
*b* = 9.4146 (2) Å	Cell parameters from 9892 reflections
*c* = 13.0721 (3) Å	θ = 3.1–28.4°
α = 75.858 (1)°	µ = 1.62 mm^−^^1^
β = 78.460 (1)°	*T* = 200 K
γ = 77.488 (1)°	Platelet, colourless
*V* = 930.75 (4) Å^3^	0.60 × 0.44 × 0.17 mm

### Data collection

**Table:**

Bruker APEXII CCD diffractometer	4644 independent reflections
Radiation source: sealed tube	4391 reflections with *I* > 2σ(*I*)
Graphite monochromator	*R*_int_ = 0.015
Detector resolution: 8.3333 pixels mm^-1^	θ_max_ = 28.4°, θ_min_ = 2.5°
φ and ω scans	*h* = −10→10
Absorption correction: numerical (SADABS; Bruker, 2011)	*k* = −12→12
*T*_min_ = 0.824, *T*_max_ = 1.000	*l* = −16→17
16101 measured reflections	

### Refinement

**Table:**

Refinement on *F*^2^	Hydrogen site location: inferred from neighbouring sites
Least-squares matrix: full	H-atom parameters constrained
*R*[*F*^2^ > 2σ(*F*^2^)] = 0.015	*w* = 1/[σ^2^(*F*_o_^2^) + (0.0111*P*)^2^ + 0.3546*P*] where *P* = (*F*_o_^2^ + 2*F*_c_^2^)/3
*wR*(*F*^2^) = 0.037	(Δ/σ)_max_ = 0.002
*S* = 1.15	Δρ_max_ = 0.28 e Å^−^^3^
4644 reflections	Δρ_min_ = −0.31 e Å^−^^3^
191 parameters	Extinction correction: SHELXL2017 (Sheldrick, 2015), Fc^*^=kFc[1+0.001xFc^2^λ^3^/sin(2θ)]^-1/4^
0 restraints	Extinction coefficient: 0.0173 (7)
Primary atom site location: dual	

### Special details

**Table:**

Geometry. All esds (except the esd in the dihedral angle between two l.s. planes) are estimated using the full covariance matrix. The cell esds are taken into account individually in the estimation of esds in distances, angles and torsion angles; correlations between esds in cell parameters are only used when they are defined by crystal symmetry. An approximate (isotropic) treatment of cell esds is used for estimating esds involving l.s. planes.
Refinement. Carbon-bound H atoms were placed in calculated positions and were included in the refinement in the riding model approximation, with *U*(H) set to 1.2 *U*_eq_(C).Two reflections with large differences between their observed and calculated intensity were omitted. This is probably due to obstruction by the beam stop.

### Fractional atomic coordinates and isotropic or equivalent isotropic displacement parameters (Å^2^)

**Table:**

	*x*	*y*	*z*	*U*_iso_*/*U*_eq_	
Cd1	0.89235 (2)	−0.00613 (2)	0.63560 (2)	0.02744 (4)	
S11	0.78076 (5)	−0.22154 (4)	0.60114 (3)	0.03471 (8)	
S12	0.78142 (4)	0.06086 (3)	0.43671 (3)	0.02551 (7)	
S21	0.84065 (5)	−0.02186 (4)	0.84095 (3)	0.03026 (7)	
S22	0.80123 (4)	0.25713 (4)	0.67413 (2)	0.02688 (7)	
N1	0.66681 (14)	−0.17652 (12)	0.41751 (9)	0.0263 (2)	
N2	0.76538 (14)	0.25083 (12)	0.88135 (8)	0.0252 (2)	
C11	0.73731 (15)	−0.12060 (14)	0.47817 (10)	0.0234 (2)	
C12	0.61897 (18)	−0.32572 (16)	0.45381 (11)	0.0323 (3)	
H12A	0.522086	−0.328088	0.418787	0.039*	
H12B	0.579076	−0.343192	0.531802	0.039*	
C13	0.7621 (2)	−0.44839 (16)	0.43028 (13)	0.0402 (3)	
H13	0.865773	−0.457570	0.456951	0.048*	
C14	0.7529 (3)	−0.54414 (18)	0.37491 (16)	0.0535 (5)	
H14A	0.650843	−0.537598	0.347257	0.064*	
H14B	0.848334	−0.620204	0.362346	0.064*	
C15	0.6278 (2)	−0.09675 (16)	0.31078 (11)	0.0342 (3)	
H15A	0.641253	0.008254	0.299629	0.041*	
H15B	0.506864	−0.097581	0.307348	0.041*	
C16	0.74035 (18)	−0.16349 (18)	0.22396 (11)	0.0367 (3)	
H16	0.860609	−0.180714	0.223617	0.044*	
C17	0.6869 (2)	−0.2001 (2)	0.14846 (13)	0.0468 (4)	
H17A	0.567516	−0.184585	0.146136	0.056*	
H17B	0.767061	−0.242477	0.095478	0.056*	
C21	0.79907 (15)	0.16963 (13)	0.80674 (10)	0.0221 (2)	
C22	0.78373 (18)	0.18663 (15)	0.99407 (10)	0.0302 (3)	
H22A	0.861484	0.238077	1.015705	0.036*	
H22B	0.836930	0.080256	1.001404	0.036*	
C23	0.6172 (2)	0.19957 (18)	1.06666 (12)	0.0420 (4)	
H23	0.528018	0.158623	1.053323	0.050*	
C24	0.5865 (3)	0.2647 (2)	1.14844 (14)	0.0635 (6)	
H24A	0.673453	0.306597	1.163574	0.076*	
H24B	0.477492	0.269906	1.192382	0.076*	
C25	0.72097 (18)	0.41463 (14)	0.85522 (11)	0.0307 (3)	
H25A	0.640375	0.449151	0.915541	0.037*	
H25B	0.662323	0.445861	0.791634	0.037*	
C26	0.8754 (2)	0.48598 (16)	0.83349 (13)	0.0407 (3)	
H26	0.964495	0.461004	0.777955	0.049*	
C27	0.8956 (3)	0.5809 (2)	0.88618 (19)	0.0629 (5)	
H27A	0.808789	0.608066	0.942160	0.075*	
H27B	0.997242	0.622596	0.868496	0.075*	

### Atomic displacement parameters (Å^2^)

**Table:**

	*U*^11^	*U*^22^	*U*^33^	*U*^12^	*U*^13^	*U*^23^
Cd1	0.02830 (6)	0.03364 (6)	0.02419 (6)	−0.00931 (4)	−0.00001 (4)	−0.01311 (4)
S11	0.0509 (2)	0.03708 (18)	0.02178 (15)	−0.02237 (16)	−0.00800 (14)	−0.00194 (13)
S12	0.02576 (15)	0.02429 (14)	0.02685 (15)	−0.00332 (11)	−0.00321 (12)	−0.00771 (11)
S21	0.04427 (19)	0.02305 (14)	0.02257 (15)	−0.00670 (13)	−0.00156 (13)	−0.00538 (11)
S22	0.03366 (16)	0.02749 (15)	0.02034 (14)	−0.00547 (12)	−0.00683 (12)	−0.00441 (11)
N1	0.0255 (5)	0.0302 (5)	0.0262 (5)	−0.0061 (4)	−0.0051 (4)	−0.0093 (4)
N2	0.0296 (5)	0.0239 (5)	0.0214 (5)	0.0007 (4)	−0.0052 (4)	−0.0070 (4)
C11	0.0198 (5)	0.0288 (6)	0.0225 (5)	−0.0051 (4)	0.0005 (4)	−0.0093 (5)
C12	0.0311 (7)	0.0389 (7)	0.0328 (7)	−0.0180 (6)	−0.0036 (5)	−0.0096 (6)
C13	0.0388 (8)	0.0292 (7)	0.0493 (9)	−0.0108 (6)	−0.0098 (7)	0.0045 (6)
C14	0.0623 (11)	0.0312 (8)	0.0604 (11)	−0.0166 (7)	0.0138 (9)	−0.0086 (7)
C15	0.0404 (8)	0.0328 (7)	0.0330 (7)	0.0007 (6)	−0.0179 (6)	−0.0098 (6)
C16	0.0267 (6)	0.0527 (9)	0.0279 (7)	−0.0097 (6)	−0.0037 (5)	−0.0010 (6)
C17	0.0537 (10)	0.0539 (10)	0.0336 (8)	−0.0058 (8)	−0.0040 (7)	−0.0163 (7)
C21	0.0188 (5)	0.0252 (6)	0.0227 (5)	−0.0039 (4)	−0.0028 (4)	−0.0064 (4)
C22	0.0365 (7)	0.0318 (6)	0.0212 (6)	0.0017 (5)	−0.0062 (5)	−0.0089 (5)
C23	0.0427 (8)	0.0414 (8)	0.0309 (7)	−0.0007 (7)	0.0002 (6)	0.0023 (6)
C24	0.0823 (14)	0.0469 (10)	0.0345 (9)	0.0170 (10)	0.0156 (9)	−0.0033 (7)
C25	0.0376 (7)	0.0243 (6)	0.0286 (6)	0.0035 (5)	−0.0072 (5)	−0.0091 (5)
C26	0.0487 (9)	0.0285 (7)	0.0445 (9)	−0.0070 (6)	−0.0074 (7)	−0.0067 (6)
C27	0.0769 (14)	0.0452 (10)	0.0796 (14)	−0.0149 (9)	−0.0308 (12)	−0.0187 (10)

### Geometric parameters (Å, º)

**Table:**

Cd1—S11	2.5558 (3)	C15—C16	1.483 (2)
Cd1—S22	2.5709 (3)	C15—H15A	0.9900
Cd1—S12^i^	2.6021 (3)	C15—H15B	0.9900
Cd1—S21	2.6050 (3)	C16—C17	1.297 (2)
Cd1—S12	2.8016 (3)	C16—H16	0.9500
S11—C11	1.7162 (13)	C17—H17A	0.9500
S12—C11	1.7532 (13)	C17—H17B	0.9500
S21—C21	1.7224 (12)	C22—C23	1.484 (2)
S22—C21	1.7263 (12)	C22—H22A	0.9900
N1—C11	1.3213 (16)	C22—H22B	0.9900
N1—C15	1.4735 (17)	C23—C24	1.315 (3)
N1—C12	1.4779 (17)	C23—H23	0.9500
N2—C21	1.3333 (15)	C24—H24A	0.9500
N2—C22	1.4738 (16)	C24—H24B	0.9500
N2—C25	1.4749 (16)	C25—C26	1.490 (2)
C12—C13	1.490 (2)	C25—H25A	0.9900
C12—H12A	0.9900	C25—H25B	0.9900
C12—H12B	0.9900	C26—C27	1.307 (2)
C13—C14	1.308 (2)	C26—H26	0.9500
C13—H13	0.9500	C27—H27A	0.9500
C14—H14A	0.9500	C27—H27B	0.9500
C14—H14B	0.9500		
			
S11—Cd1—S22	143.705 (13)	N1—C15—H15A	109.1
S11—Cd1—S12^i^	103.129 (12)	C16—C15—H15A	109.1
S22—Cd1—S12^i^	111.289 (11)	N1—C15—H15B	109.1
S11—Cd1—S21	108.203 (11)	C16—C15—H15B	109.1
S22—Cd1—S21	70.264 (10)	H15A—C15—H15B	107.8
S12^i^—Cd1—S21	110.826 (11)	C17—C16—C15	124.89 (14)
S11—Cd1—S12	67.486 (10)	C17—C16—H16	117.6
S22—Cd1—S12	96.950 (10)	C15—C16—H16	117.6
S12^i^—Cd1—S12	96.257 (9)	C16—C17—H17A	120.0
S21—Cd1—S12	152.651 (11)	C16—C17—H17B	120.0
C11—S11—Cd1	91.26 (4)	H17A—C17—H17B	120.0
C11—S12—Cd1^i^	100.48 (4)	N2—C21—S21	120.81 (9)
C11—S12—Cd1	82.68 (4)	N2—C21—S22	119.72 (9)
Cd1^i^—S12—Cd1	83.743 (9)	S21—C21—S22	119.47 (7)
C21—S21—Cd1	84.58 (4)	N2—C22—C23	112.57 (11)
C21—S22—Cd1	85.57 (4)	N2—C22—H22A	109.1
C11—N1—C15	123.59 (11)	C23—C22—H22A	109.1
C11—N1—C12	121.48 (11)	N2—C22—H22B	109.1
C15—N1—C12	114.93 (11)	C23—C22—H22B	109.1
C21—N2—C22	123.16 (10)	H22A—C22—H22B	107.8
C21—N2—C25	122.11 (11)	C24—C23—C22	123.46 (19)
C22—N2—C25	114.53 (10)	C24—C23—H23	118.3
N1—C11—S11	120.74 (10)	C22—C23—H23	118.3
N1—C11—S12	120.64 (10)	C23—C24—H24A	120.0
S11—C11—S12	118.58 (7)	C23—C24—H24B	120.0
N1—C12—C13	113.50 (11)	H24A—C24—H24B	120.0
N1—C12—H12A	108.9	N2—C25—C26	111.95 (11)
C13—C12—H12A	108.9	N2—C25—H25A	109.2
N1—C12—H12B	108.9	C26—C25—H25A	109.2
C13—C12—H12B	108.9	N2—C25—H25B	109.2
H12A—C12—H12B	107.7	C26—C25—H25B	109.2
C14—C13—C12	123.61 (16)	H25A—C25—H25B	107.9
C14—C13—H13	118.2	C27—C26—C25	123.99 (18)
C12—C13—H13	118.2	C27—C26—H26	118.0
C13—C14—H14A	120.0	C25—C26—H26	118.0
C13—C14—H14B	120.0	C26—C27—H27A	120.0
H14A—C14—H14B	120.0	C26—C27—H27B	120.0
N1—C15—C16	112.59 (12)	H27A—C27—H27B	120.0
			
C15—N1—C11—S11	−179.31 (10)	N1—C15—C16—C17	128.82 (17)
C12—N1—C11—S11	1.48 (17)	C22—N2—C21—S21	8.51 (17)
C15—N1—C11—S12	2.97 (17)	C25—N2—C21—S21	−176.94 (10)
C12—N1—C11—S12	−176.24 (9)	C22—N2—C21—S22	−171.47 (10)
Cd1—S11—C11—N1	−178.09 (10)	C25—N2—C21—S22	3.08 (17)
Cd1—S11—C11—S12	−0.33 (7)	Cd1—S21—C21—N2	−176.69 (10)
Cd1^i^—S12—C11—N1	−99.72 (10)	Cd1—S21—C21—S22	3.29 (6)
Cd1—S12—C11—N1	178.07 (10)	Cd1—S22—C21—N2	176.65 (10)
Cd1^i^—S12—C11—S11	82.51 (7)	Cd1—S22—C21—S21	−3.33 (7)
Cd1—S12—C11—S11	0.30 (6)	C21—N2—C22—C23	−114.38 (14)
C11—N1—C12—C13	−86.54 (16)	C25—N2—C22—C23	70.69 (16)
C15—N1—C12—C13	94.18 (15)	N2—C22—C23—C24	−123.84 (16)
N1—C12—C13—C14	−123.54 (16)	C21—N2—C25—C26	−91.28 (15)
C11—N1—C15—C16	110.28 (15)	C22—N2—C25—C26	83.71 (15)
C12—N1—C15—C16	−70.47 (16)	N2—C25—C26—C27	−122.19 (18)

Symmetry code: (i) −*x*+2, −*y*, −*z*+1.

### Hydrogen-bond geometry (Å, º)

**Table:**

*D*—H···*A*	*D*—H	H···*A*	*D*···*A*	*D*—H···*A*
C12—H12*B*···S11	0.99	2.59	2.9783 (14)	103
C15—H15*A*···S12	0.99	2.50	3.0438 (14)	115
C22—H22*B*···S21	0.99	2.50	3.0381 (13)	114
C25—H25*B*···S22	0.99	2.56	2.9845 (14)	106
